# Metabolic Shift in Porcine Spermatozoa during Sperm Capacitation-Induced Zinc Flux

**DOI:** 10.3390/ijms25147919

**Published:** 2024-07-19

**Authors:** Tyler Weide, Kayla Mills, Ian Shofner, Matthew W. Breitzman, Karl Kerns

**Affiliations:** 1 Department of Animal Science, Iowa State University, Ames, IA 50011, USA; tjweide@iastate.edu (T.W.); ishofner@iastate.edu (I.S.); 2 U.S. Department of Agriculture, Agricultural Research Service, Beltsville Agricultural Research Center (BARC), Beltsville, MD 20705, USA; kayla.mills@usda.gov; 3 W.M. Keck Metabolomics Research Laboratory, Iowa State University, Ames, IA 50011, USA; breitzma@iastate.edu

**Keywords:** spermatozoa, capacitation, metabolomics, metabolites, glycolysis, image-based flow cytometry

## Abstract

Mammalian spermatozoa rely on glycolysis and mitochondrial oxidative phosphorylation for energy leading up to fertilization. Sperm capacitation involves a series of well-regulated biochemical steps that are necessary to give spermatozoa the ability to fertilize the oocyte. Additionally, zinc ion (Zn^2+^) fluxes have recently been shown to occur during mammalian sperm capacitation. Semen from seven commercial boars was collected and analyzed using image-based flow cytometry before, after, and with the inclusion of 2 mM Zn^2+^ containing in vitro capacitation (IVC) media. Metabolites were extracted and analyzed via Gas Chromatography-Mass Spectrometry (GC-MS), identifying 175 metabolites, with 79 differentially abundant across treatments (*p* < 0.05). Non-capacitated samples showed high levels of respiration-associated metabolites including glucose, fructose, citric acid, and pyruvic acid. After 4 h IVC, these metabolites significantly decreased, while phosphate, lactic acid, and glucitol increased (*p* < 0.05). With zinc inclusion, we observed an increase in metabolites such as lactic acid, glucitol, glucose, fructose, myo-inositol, citric acid, and succinic acid, while saturated fatty acids including palmitic, dodecanoic, and myristic acid decreased compared to 4 h IVC, indicating regulatory shifts in metabolic pathways and fatty acid composition during capacitation. These findings underscore the importance of metabolic changes in improving artificial insemination and fertility treatments in livestock and humans.

## 1. Introduction

Sperm capacitation is an important physiological process that prepares mammalian spermatozoa for fertilization. Initially described in independent reporting from Chang and Austin in 1951 [1,2], sperm capacitation encompasses a series of well-regulated biochemical steps, signaling pathways, and physiological outcomes that prepare the sperm cell to successfully fertilize the oocyte. Despite its long recognition, the complex regulatory and metabolic pathways governing this process are not entirely understood.

After entry into the female genital tract and after reaching the oviductal sperm reservoir, there is a substantial influx of calcium and bicarbonate ions in response to a newly ovulated oocyte, which is crucial for activating signaling pathways within spermatozoa [3,4]. Intracellular pH is increased during sperm capacitation primarily by the influx of bicarbonate ions, as well as Ca^2+^ ions, which leads to protein kinase A (PKA) activation [5]. Intracellular HCO^3−^ and Ca^2+^ ions bind to the soluble adenylate cyclase (sAC), which catalyzes adenosine triphosphate to adenosine monophosphate, activating PKA [4,5]. Increased PKA activity leads to tyrosine phosphorylation of proteins located on the sperm flagellum, plasma membrane, equatorial segment, and acrosomal region, inducing capacitation and hyperactivation in spermatozoa [6]. It has also been shown in vitro that capacitation is associated with the removal of cholesterol from the plasma membrane [7]. The activation of PKA in the sperm cell increases membrane fluidity following cholesterol efflux [8,9].

The optimal pH for normal sperm function and metabolism is around 7.2–7.8 in humans [10] and ranges between 6.9 and 7.2 in boars [11]. Homeostasis of intracellular pH is an important process for sperm cell function as the female genital tract pH ranges from 4.5 to 7.5 in mammals, with the pH increasing as the sperm cell travels closer to the site of fertilization [10].

As the sperm becomes closer to the site of fertilization, the influx of calcium ions causes a wave of vesiculation across the sperm head, destabilizing the acrosomal vesicles and releasing the contents in the acrosomal matrix, an event known as acrosomal exocytosis [12]. The acrosomal matrix and the inner acrosomal membrane contain proteins and enzymes necessary to bind to the zona pellucida and penetrate the egg, rendering the sperm competent to fertilize [13]. Acrosomal exocytosis is considered a pre-requisite to fertilization and is a hallmark of capacitation [14].

These well-regulated biochemical steps and cellular modifications of spermatozoa, known as sperm capacitation, are energy intensive and require energy produced from two main metabolic pathways: oxidative phosphorylation and anaerobic glycolysis [15]. While mammalian sperm contain the necessary machinery to support the energy demands for motility and capacitation from glycolysis and mitochondrial oxidative phosphorylation, ejaculated sperm from humans and mice showcase the ability to utilize non-glycolytic energy sources such as pyruvate and citrate [16].

Recently, a study has shown that zinc efflux appears to play an important role in regulating sperm capacitation pathways by activating zinc-containing enzymes and proteases involved in sperm penetration of the zona pellucida [17]. Since then, 1752 zinc-interacting proteins have been identified [18]. With respect to these developments, this study focuses on evaluating the sperm metabolome of boars before and after in vitro capacitation along with the inclusion of Zn^2+^. By utilizing Gas Chromatography-Mass Spectrometry (GC-MS) analysis, we aim to identify metabolites changing before and after capacitation and to observe the impact of Zn^2+^ inclusion on capacitation events. This study aims to contribute to our understanding of the metabolic changes in capacitation events and has potential impacts for enhancing or reducing fertilization outcomes in both livestock and human reproductive medicine, depending on desired outcomes.

## 2. Results

**Figure 1:**
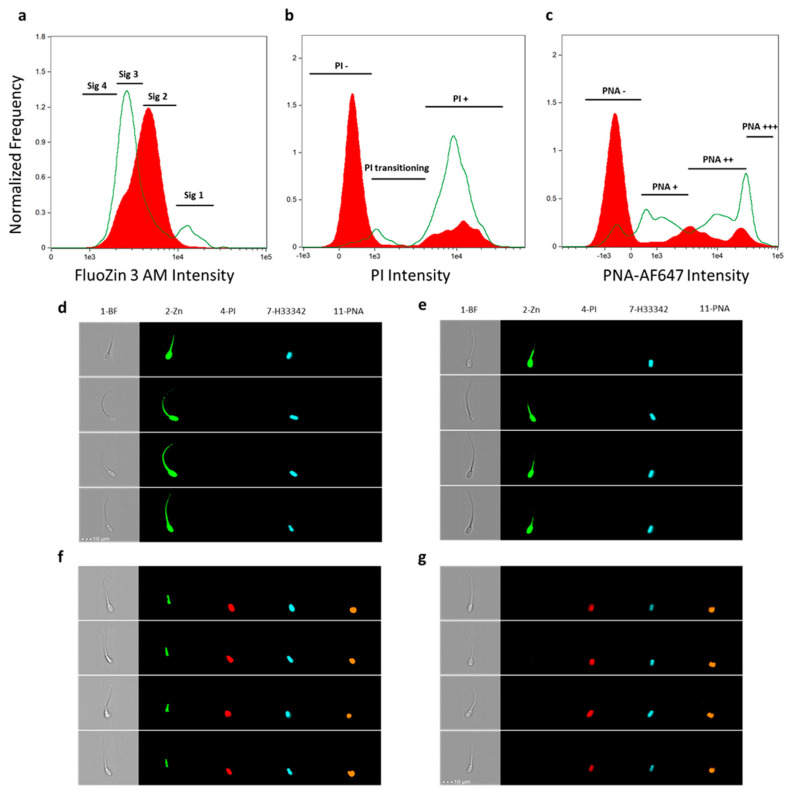
Image-based flow cytometry validation of in vitro capacitation. (**a**–**c**) Histogram overlays between 0 h (shown in red, filled in solid) and 4 h IVC (shown in green, not filled in), showing changes in the fluorescence of biomarkers between the two treatments. (**a**) FluoZin 3 AM intensity reporting zinc ion localization, (**b**) PI intensity reflecting plasma membrane integrity/remodeling, and (**c**) lectin PNA conjugated to AlexaFluor 647 intensity reflecting acrosomal integrity/remodeling. Boar #2 is displayed here for illustrative purposes. (**d**–**g**) Images taken from the Cytek^®^ Amnis^®^ ImageStream^®X^ MkII image-based flow cytometer (Fremont, CA, USA) validating capacitation status. Columns are denoted by channel number and fluorescent probe/target, and rows are single-cell images of different spermatozoa. Channels used: (1) Brightfield (BF), (2) FluoZin 3 AM reflecting zinc (Zn) ion localization, (4) propidium iodide (PI), (7) Hoescht 33342 nuclear stain, and (11) lectin peanut agglutin (PNA)-Alexa Fluor 647™. Examples of (**d**) zinc signature 1 spermatozoa, (**e**) zinc signature 2, (**f**) zinc signature 3, and (**g**) zinc signature 4. Total percentages of sperm per biomarker classification are in Table S1.

In this study, image-based flow cytometry (IBFC) was utilized to assess the capacitation status of boar spermatozoa (Figure 1). Shifts in sperm zinc signature classification after four hours of in vitro capacitation were observed, as previously reported [17]. The IBFC images show spermatozoa before and after 4 h of in vitro capacitation (IVC). Brightfield imaging (Channel 1-BF) provides a clear view of sperm morphology, ensuring only structurally intact cells were analyzed. The FluoZin 3 AM zinc probe (Channel 2-Zn) indicated zinc ion localization patterns, which have been previously correlated with sperm capacitation states [17,19]. Percentages of Zinc signatures can be found in Table S1. Notably, FluoZin 3 AM fluorescence shifted from zinc signature 1 and 2 before in vitro capacitation to zinc signature 3 and 4 after four hours of IVC. Propidium iodide staining (Channel 4-PI), which permeates only cells with compromised plasma membranes, was used to assess plasma membrane integrity. The presence of PI-stained cells post-IVC indicates changes in sperm plasma membrane integrity/capacitation-induced remodeling, which is required for sperm capacitation [20]. Percentages of PI stained cells can be found in Table S3. Hoechst 33342 (Channel 7-H33342) was used to stain and visualize the sperm nucleus. Finally, lectin peanut agglutinin (PNA) conjugated to AlexaFluor 647™ (Channel 11-PNA) assesses acrosomal integrity/remodeling. A noticeable shift in the PNA binding pattern from 0 h to 4 h suggests acrosomal exocytosis or remodeling has occurred, indicative of sperm capacitation [12]. Percentages of PNA stained cells can be found in Table S2 Collectively, these multi-parametric analyses confirm the capacitation status of our samples. 

Our comprehensive metabolomics analysis reveals distinct patterns of metabolite utilization associated with boar sperm capacitation. Using Gas Chromatography-Mass Spectrometry (GC-MS), we quantified the variations in metabolite concentrations across non-capacitated spermatozoa (0 h), post IVC (4 h), and post IVC supplemented with 2 mM extracellular zinc (4 h + Zn). In the initial evaluations, we observed the consumption of key sugars, which reflects the heightened energetic demands during capacitation.

**Figure 2:**
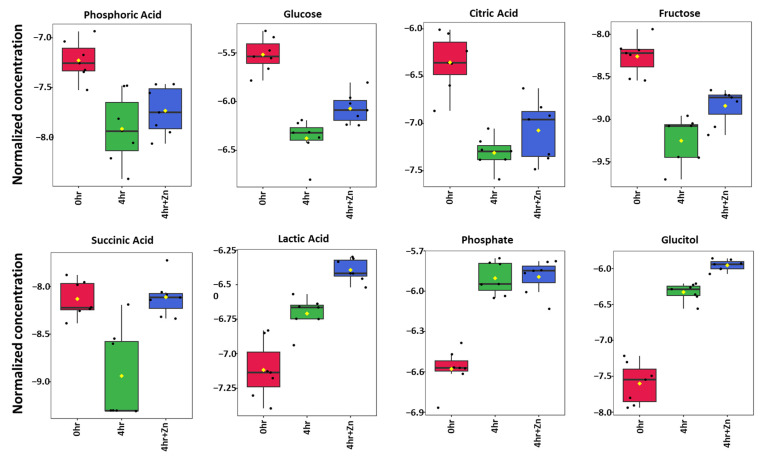
Changes in abundance in key metabolites associated with cellular respiration after in vitro capacitation. A total of 79 metabolites involved in cellular respiration pathways were identified as differentially abundant following capacitation (*p* < 0.05; complete heatmap of those identified displayed in Figure S2). Eight of the well-known metabolites that are represented here are integral to well-known cellular respiration processes. Non-capacitated (0 h) is shown on the left in red, capacitated (4 h) is shown in the middle in green, and capacitated +2 mM zinc (4 h + Zn) is shown on the right in blue, for each respective metabolite.

After 4 h of IVC, the abundance of certain metabolites such as glucitol, lactic acid, and phosphate increased (Figure 2). Conversely, key glycolytic substrates such as glucose, fructose, citric acid, succinic acid, and phosphoric acid were significantly depleted, reflecting their consumption during energy-intensive capacitation events. Not all of the reported 79 statistically significant metabolites between the three treatments are glycolytic substrates. Figure 2 represents eight of the well-known metabolites found in cellular respiration pathways. All 175 metabolites reported can be found in Supplementary Table S4.

**Figure 3:**
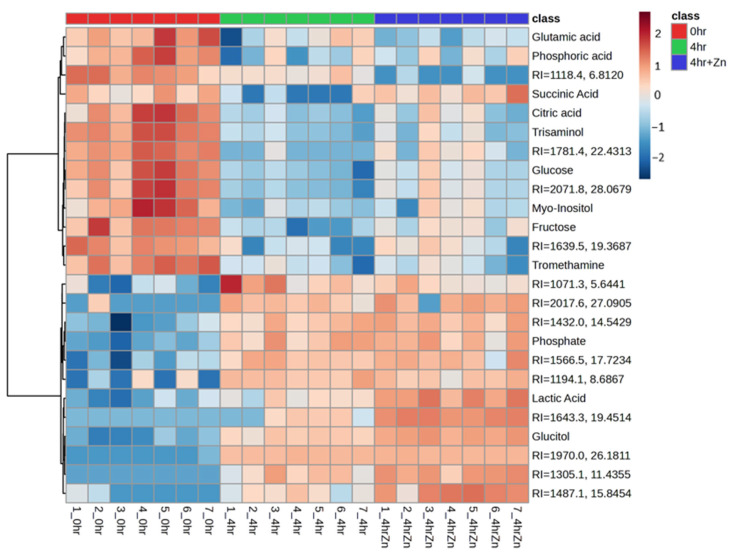
Heat map representing top 25 differentially abundant metabolites in sperm metabolome of all sires individually across treatments. Fold change is illustrated by a gradient from dark blue (negative) to dark red (positive). 0 h (non-capacitated) are in red, 4 h IVC are in green, and 4 h IVC + 2 mM zinc are in blue, by class. Metabolites starting with the name “RI=” are unidentified features that have been given a retention-index-based name for tracking purposes. Total 79 statistically significant metabolites are reported in Figure S2.

Building on the individual metabolite changes, we next sought to visualize the global metabolic variations during sperm capacitation. The top 25 differentially abundant metabolites are displayed in Figure 3. Heatmap analysis unveiled metabolic shifts, which include the relative abundance of TCA cycle intermediates and their downstream effectors, which varied distinctly between the capacitated and non-capacitated treatment groups. Furthermore, the presence of extracellular zinc appears to modulate the concentrations of several key metabolites, including succinic acid. Metabolites starting with the name “RI=” are unidentified features that have been given a retention-index and retention-time-based name for tracking purposes. The 79 statistically significant metabolites are reported in Figure S2, and the total 175 metabolites are reported in Supplementary Table S4.

**Figure 4:**
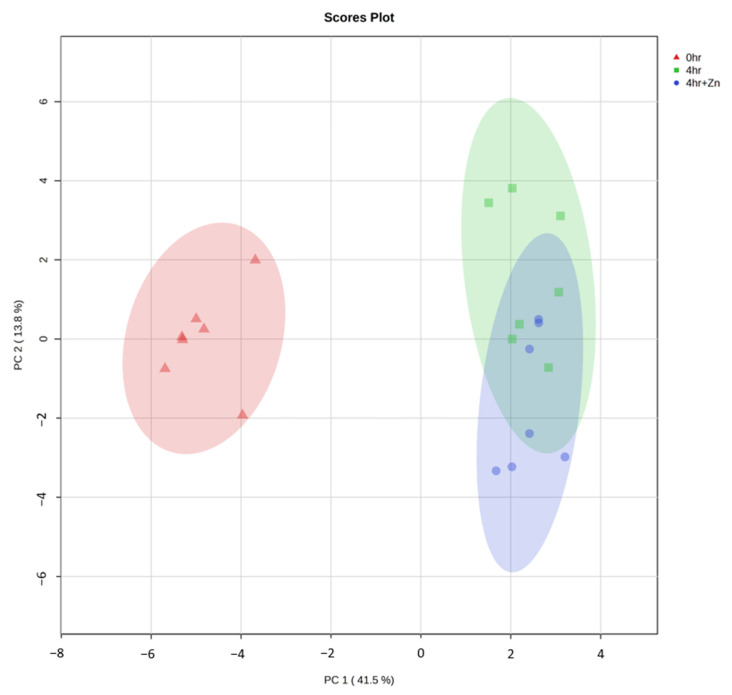
Principal component analysis. PCA analysis was employed to visualize the metabolic differentiation between non-capacitated spermatozoa (red triangles), capacitated spermatozoa (green squares), and capacitated spermatozoa supplemented with 2 mM zinc (blue circles). Each symbol represents an individual sample plotted against the first two principal components. Colored ellipses represent confidence regions, indicating the clustering of samples within each group, with each color corresponding to a different treatment.

The principal component analysis (PCA) plot in Figure 4 elucidates distinct clustering of samples corresponding to each treatment group. Non-capacitated samples (red triangles) are predominantly situated along the negative axis of PC1, forming a discrete cluster within the red ellipse. This segregation indicates a unique metabolic signature that is noticeably different from the post-capacitation states. In contrast, capacitated samples without zinc (green squares) occupy the positive PC1 axis and are encapsulated in the green ellipse. Capacitated samples supplemented with zinc (blue circles) are distributed between the non-capacitated and capacitated-without-zinc samples along the PC2 axis. This intermediate positioning reflects the modulatory impact of zinc on the sperm metabolic profile during capacitation.

**Figure 5:**
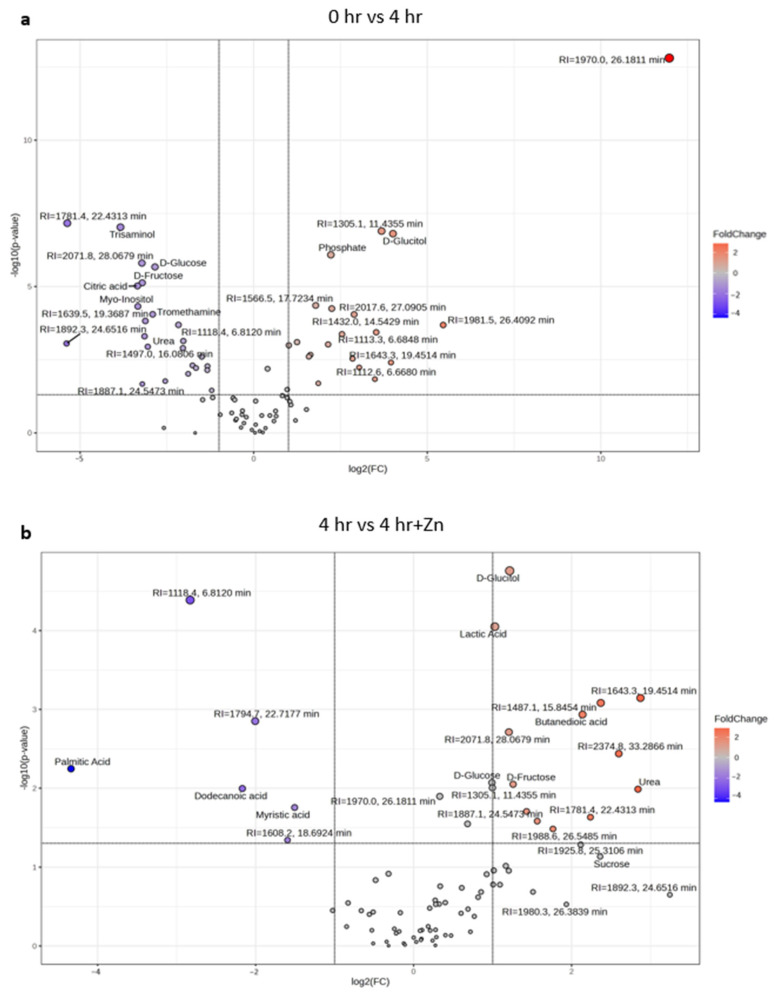
Volcano plots representing top 25 differentially abundant metabolites in sperm metabolome across treatments. Fold change (log2(FC)) is represented on the *X*-axis, and the statistical significance (−log10(*p*-value)) is represented on the *Y*-axis. Metabolites with labels are the top 25 differentially abundant within treatment groups. Varying size in circles in (**a**,**b**) represents the relative abundance of each metabolite (**a**) Comparison before and after 4 h of in vitro capacitation: 23 metabolites were less abundant and 19 metabolites were more abundant after 4 h of IVC. (**b**) Changes in sperm metabolome abundances between 4 h IVC and 4 h IVC + 2 mM Zinc: 6 metabolites were less abundant and 13 metabolites were more abundant after 4 h of IVC + 2 mM zinc inclusion. Metabolites starting with the name “RI=” are unidentified features that have been given a retention-index-based name for tracking purposes.

The volcano plots in Figure 5 provide a visualization of the metabolic shifts between the three treatments, underlying the biochemical transformation that spermatozoa undergo in preparation for successful fertilization. A volcano plot that contrasts the changes in metabolite abundance between non-capacitated (0 h) and capacitated (4 h) samples is visualized in Figure 5a. Metabolites higher in abundance after capacitation are indicated in red circles on the right side of the graph, revealing an increase in identified metabolites such as glucitol and phosphate. Conversely, the blue circles represent metabolites less abundant upon capacitation, including the identified metabolites glucose, fructose, citric acid, myo-inositol, and urea.

Changes in metabolite abundance between capacitated (4 h) and capacitated samples supplemented with 2 mM zinc (4 h + Zn) are visualized in Figure 5b. The identified metabolites that are significantly higher in abundance after zinc supplementation include glucitol, lactic acid, succinic acid, urea, fructose, and sucrose. The identified metabolites that are significantly lower in abundance after zinc inclusion are palmitic acid, dodecanoic acid, and myristic acid.

## 3. Discussion

Spermatozoa in boars heavily rely on mitochondrial oxidative phosphorylation to produce ATP for motility [21]. This pathway ensures a high yield of ATP, necessary for motility and initial stages of capacitation. While mitochondrial oxidative phosphorylation provides the bulk of energy in oxygen-rich environments, studies have shown that monosaccharides such as glucose and fructose are critical for facilitating capacitation and supporting the continued hyperactivated motility necessary for successful fertilization in humans [22]. It has also been reported that glycolysis plays a major role in supplying energy for sperm motility in mice [23]. It has been shown that glucose consumption is enhanced in media that support sperm capacitation, suggesting up-regulation of glucose metabolic pathways in mice [24], which has not been previously reported in boars. While the initial reliance on mitochondrial respiration provides a high yield of ATP in favorable oxygen-rich conditions, the dynamic interplay of both mitochondrial oxidative phosphorylation and glycolysis across mammalian species reflects the sperm cell’s ability to adapt to fluctuating environments as they progress towards the oocyte.

Our study presents an integrative examination of the metabolic alterations during sperm capacitation, a vital preparatory step for successful mammalian fertilization. Utilizing Gas Chromatography-Mass Spectrometry (GC-MS) to elucidate the sperm metabolome of commercial boars, we identified significant metabolic changes before and after capacitation, as well as with zinc supplementation.

After 4 h of IVC, the reduction in primary energy substrates such as glucose, fructose, citric acid, and phosphoric acid, as shown in Figure 1, supports the increased energy demands for sperm motility and acrosomal exocytosis preparation [25]. The observed decrease in phosphoric acid and significant increase in phosphate abundance further supports the hypothesis of high ATP utilization during capacitation by oxidative phosphorylation. The shift from aerobic respiration pathways, as indicated by the decrease in citric acid, is consistent with the spermatozoa’s reliance on the TCA cycle before capacitation, transitioning to a heightened use of ATP during capacitation, as evidenced by the decline in phosphoric acid. The utilization of citric acid as an energy source confirms the ability of boar sperm to utilize non-glycolytic substrates for the energy demands of motility and capacitation [16]. Among the identified metabolites, myo-inositol, a precursor to phosphatidylinositol polyphosphates (PIPs), which are key substrates in the signal transduction pathway in the plasma membrane [26] and are responsible for the regulation of intracellular Ca^2+^ concentration [27], showed a significant decrease after 4 h of IVC. Low concentrations of myo-inositol in spermatozoa have been associated with a significant reduction in fertility in transgenic mice [28].

The increase in substrates such as glucitol, phosphate, and lactic acid after 4 h of IVC suggests that anaerobic glycolysis plays a role in the metabolism of sperm capacitation, as previously reported by Storey [15]. We observed a substantial increase in the concentration of glucitol after 4 h of IVC. This polyol is implicated in cellular osmoregulation and may provide a protective mechanism against the hyperosmotic conditions spermatozoa encounter during the capacitation process [29]. Phosphate showed an increase in concentration after 4 h of IVC, which is consistent with the heightened ATP turnover expected during the energy-intensive phase of capacitation. Lactic acid also showed an increase in concentration after 4 h of IVC, suggesting an increased reliance on anaerobic glycolysis. Studies in bulls suggest that highly fertile animals with a higher abundance of lactic acid post-capacitation utilize anaerobic glycolysis more efficiently than low-fertility animals with a lower abundance of lactic acid [30].

The inclusion of zinc in our study adds a novel dimension in metabolic regulation, demonstrating its potential role in modulating the sperm capacitation process. The distinct metabolic profile of spermatozoa in the presence of extracellular zinc, featuring elevated levels of glucitol, lactic acid, succinic acid, urea, fructose, and sucrose compared to non-zinc-supplemented capacitation, suggests that zinc has a regulatory influence on metabolic function and cellular respiration during sperm capacitation. Furthermore, saturated fatty acids such as palmitic acid (C16:0), dodecanoic acid (C12:0), and myristic acid (C14:0) all showed significantly lower abundance in the 4 h IVC + zinc treatments but not in the other two treatments, further indicating some regulatory role of fatty acid composition during capacitation events. Palmitic acid in spermatozoa was previously found to have a positive correlation to total sperm count and is one of the main saturated fatty acids in mammalian sperm cells, indicating its importance in sperm production and fertilization [31]. Extensive research has established zinc as a pivotal regulator within the biochemical pathways governing sperm capacitation, enzymatic function, and subsequent fertilization processes [17].

## 4. Materials and Methods

### 4.1. Experimental Design

To evaluate metabolic shifts during sperm capacitation in boars, three treatments were compared: non-capacitated (0 h), capacitated (4 h), and capacitated with 2 mM extracellular zinc (4 h + Zn). This design aimed to elucidate metabolic pathways and the regulatory role of zinc in sperm capacitation. Semen samples from seven commercial Duroc boars were included in the study. Samples were evaluated for their capacitation state by image-based flow cytometry and GC-MS-based metabolomics analysis was performed to identify significant metabolic changes.

### 4.2. Semen Collection and Processing

Seven commercial Duroc boars with known fertility from the same private boar stud were used in this study. The boars were housed one boar per pen, collected once per seven day period, and fed a commercial boar diet to maintain weight. Semen doses in this study were in excess of industry production following established standard operating procedures and were not directly collected for this research. Boar semen was collected using the standard two-gloved hand technique. Only ejaculates with >80% motility were used. Sperm concentration was determined using a hemocytometer. The semen was immediately extended (e.g., <5 min) a total of 5 times in a Preserve Xtreme extender, with the extender being within 2 °C of the semen, and delivered to Iowa State University the same day. Upon arrival, the temperature of the samples was annotated and they were stored at 17 °C. The semen was then analyzed within 24 h of collection for concentration and motility using Computer-Assisted Semen Analysis (CASA) and adjusted for a final concentration of 10 million cells/mL for each boar.

### 4.3. Computer-Assisted Semen Analysis (CASA)

For concentration and motility, a 1000 µL aliquot of each sample was placed in a 1.5 mL Eppendorf tube and warmed for 10 min at 37 °C. Samples were then mixed thoroughly to ensure even distribution of cells, and then 3.5 µL of each aliquot was loaded on to a 20 µm disposable counting chamber from Minitube (Minitüb GmbH, Tiefenbach, Germany). Concentration and motility were then assessed using a Zeiss Axioscope 5 microscope (Carl Zeiss Microscopy, LLC, Oberkochen, Germany) fitted with a Basler ace ac2440-75uc camera (Basler AG, Ahrensburg, Germany) and a 10×/0.25 A-Plan objective lens using MinitubeAndroVision^®^ software (Reference Module: 12,500/1000, Tiefenbach, Germany) (https://www.minitube.com/catalog/en/androvision-software-module-automorph-p1159/?search=1, accessed on 15 July 2024). The condenser with aperture diaphragm was set at 1 and the 6-position filter wheel was set at 2.

### 4.4. Preparation of Porcine Non-Capacitating Media and Porcine Capacitating Media

#### 4.4.1. Porcine Non-Capacitation Media (pNCM)

Non-capacitation media containing NaCl, KCl, NaH2PO_4_, Na lactate, MgCl_2_-6H_2_O, TL-HEPES, Na-pyruvate, sorbitol, gentamicin, penicillin G, polyvinyl alcohol (PVA), and glucose was prepared in 1000 mL of deionized water (ddH_2_O), as previously described [17]. Vacuum filtration was performed into an appropriate container, and the pH of the solution was adjusted to 7.20 ± 0.02 and stored at 4 °C until use.

#### 4.4.2. Porcine Capacitation Media (pCM)

Capacitation media were prepared on the same day of experiments by adding NaHCO_3_, pyruvic acid, CaCl_2,_ and bovine serum albumin to 50 mL of the prepared pNCM, as previously described [17].

#### 4.4.3. Capacitation Treatments

This study involves three different capacitation treatments: non-capacitated (0 h), capacitated (4 h), and capacitated with 2 mM extracellular zinc (4 h + Zn). A total of 20 million sperm cells were used per treatment. Non-capacitated, 0 h samples were prepared by centrifuging spermatozoa in a swinging hinge rotor centrifuge (110× *g* for 10 min), removing the supernatant, and resuspending them with pNCM. A total of 5 million spermatozoa were aliquoted for 0 h flow cytometry staining, and the remaining 100 million spermatozoa were centrifuged, the supernatant was removed, and the pellets were frozen at −80 °C until metabolomic extraction. Capacitated (4 h and 4 h + Zn) samples were washed of seminal plasma and extender was applied to 0 h samples; then, they were incubated in pCM (with and without 2 mM ZnCl_2_) for 4 h at 37 °C, as previously described [17]. At the conclusion of 4 h, the sperm were centrifuged and washed 1× with pNCM; 5 million sperm were aliquoted for IBFC and centrifuged, the supernatant was removed, and the remaining pellets were frozen at −80 °C until metabolomic extraction.

### 4.5. Image-Based Flow Cytometry

#### 4.5.1. Reagents

FluoZin™-3 (FZ3; zinc probe) from Invitrogen (2530139, Waltham, MA, USA) was reconstituted with DMSO to a stock solution of 500 µM. Lectin PNA (*A. hypogea*/peanut agglutinin) conjugated to Alexa Fluor™ 647 from Invitrogen (L32460) was reconstituted with ddH_2_O at a stock solution of 0.5 µg/mL. Hoechst 33342 (H33342) from Calbiochem (382065, San Diego, CA, USA) was reconstituted with ddH_2_O to a stock solution of 18 mM. Propidium iodide from Acros Organics (AC440300010, Geel, Belgium) was reconstituted with ddH_2_O to a stock solution of 1 mg mL^−1^.

#### 4.5.2. Flow Cytometry Probe Staining and Incubation

A total of 5 million cells per sample were centrifuged at 110× *g* for 5 min and resuspended with 100 µL of pNCM containing the following concentration of fluorescent probes diluted from the above stocks: H33342 (1:1000), propidium iodide (1:1000), FZ3 (1:500), and PNA-AF647 (1:2000). The next samples were incubated for 30 min at room temperature in the dark. The samples then had their probes removed by centrifuging, the supernatant was removed, and they were resuspended with 100 µL of phosphate-buffered saline (PBS) without NaN_3_. The samples were then incubated at 37 °C for 30 min in the dark to allow complete de-esterification of FZ3 intracellular acetoxymethyl (AM) esters. Finally, samples were run on the image-based flow cytometer.

#### 4.5.3. Image-Based Flow Cytometry (IFBC) Data Acquisition

IBFC data acquisition was performed using the ImageStream Mk II Image-Based flow cytometer, specifically a Cytek^®^ Amnis^®^ ImageStream^®X^ MkII Image-Based flow cytometer (ISX, Fremont, CA, USA) fitted with a 40× microscope objective with an imaging rate up to 2000 events/s. The sheath fluid was a phosphate-buffered solution with a pH of 7.20. The flow-core diameter and speed were 6 μm and 66 mm/s, respectively. Raw image data were acquired using INSPIRE^®^ software version 3.0. In INSPIRE^®^ data acquisition software, one bright-field channel was collected (channel 1), as was one FZ3 image (channel 2), one PI image (channel 4), one side scatter (SSC) image (channel 6), one H33342 image (channel 7), and one PNA-AF647 image (channel 11). The following lasers and power settings were used—405  nm (to excite H33342): 10  mW; 488  nm (to excite FZ3): 60  mW; 561  nm (to excite PI): 40  mW, 642  nm (to excite PNA-AF647): 25  mW; and 785  nM SSC laser: 10  mW. SpeedBeads were used to ensure focus.

#### 4.5.4. Image-Based Flow Cytometry Data Analysis

Data were analyzed using IDEAS^®^ analysis software version 6.4. The gating approach used standard focus and single-cell gating calculations created by IDEAS^®^ software. To clean up data for analysis, the Feature Finder function was used to discover image-based calculations to discard spermatozoa laterally aligned with the camera, as opposed to anteriorly/posteriorly aligned, as previously described [17].

### 4.6. Metabolomics Analysis

#### 4.6.1. Non-Targeted Metabolomic Single-Phase Extraction

Boar sperm samples were submitted to the Iowa State University W.M. Keck Metabolomics Research Laboratory (RRID:SCR_017911) for non-targeted metabolomic analysis. Sample preparation was conducted using a modified version of the methanolic extraction and sample preparation methods established by A et al. [32]. A total of 100 mg of sample was spiked with 2 µL of each of the following internal standards: nonadecanoic acid (1 mg/mL in hexane) (Sigma-Aldrich Co., St. Louis, MO, USA), ribitol (1 mg/mL in water) (Sigma-Aldrich Co., St. Louis, MO, USA), and 1,2-dilauroyl-sn-glycero-3-phosphoethanolamine (0.5 mg/mL in methanol) (Avanti Polar Lipids, Birmingham, AL, USA). The extraction was initiated with the addition of 100 µL of 80% ice-cold, LC-MS-grade methanol with 20% LC-MS-grade water (Fisher Scientific, Waltham, MA, USA). Samples were then vortexed for 30 s and placed into an ice-cold sonication water bath for 10 min at full output power. Next, the samples were vortexed for 30 s and left on ice for 30 min. The samples were then centrifuged for 7 min at maximum speed (16,000× *g*) and the supernatants were recovered. Finally, the remaining insoluble pellets were re-extracted with an additional volume of 100 µL of 80% ice-cold methanol.

#### 4.6.2. Non-Targeted Gas Chromatography-Mass Spectrometry (GC-MS) Analysis

Sixty microliters of the combined extracts were dried using a speed-vac concentrator for 10 h prior to derivation [33]. The samples were derivatized with 50 μL of methoxyamine hydrochloride (20 mg/mL in pyridine) initially added to the dried extracts followed by a 1.5 h incubation at 30 °C. Subsequently, timethylsilylation (TMS) was performed by the addition of 70 μL of bis-trimethyl silyl trifluoroacetamide with 1% Trimethylchlorosilane (BSTFA + 1% TMCS) for 30 min at 60 °C. Methoxamine HCl and BSTFA + 1% TMCS were obtained from Sigma-Aldrich, Inc. (St. Louis, MO, USA).

GC-MS analyses were performed with an Agilent 6890 gas chromatograph coupled to a model 5973 Mass Selective Detector (Agilent Technologies, Santa Clara, CA, USA). The column used was HP-5MSI 5% Phenyl Methyl Silox with 30 m × 250 µM × 0.25 µm film thickness (Agilent Technologies). One microliter of sample was injected with the inlet operating in splitless mode and held at a constant temperature of 280 °C. The oven temperature was programmed as follows: an initial temperature of 70 °C was increased to 320 °C at 5 °C/min and held for 8 min. Helium was used as a carrier gas at a flow rate of 1 mL/min. The MS transfer line was held at 280 °C. Mass Spectrometry detection was performed using electron ionization at 70 eV and the source temperature and quadrupole temperature were set at 230 °C and 150 °C, respectively. The mass data were collected in the range from *m*/*z* 40 to *m*/*z* 800. Identification and quantification were conducted using AMDIS (Automated Mass spectral Deconvolution and Identification System, National Institute of Standards and Technology (Gaithersburg, MD, USA)) with a manually curated, retention-indexed GC-MS library, with additional identification performed using the NIST20 and Wiley 11 GC-MS spectral library (Agilent Technologies, Santa Clara, CA, USA). Final quantification was calculated by integrating the corresponding peak areas relative to the area of the internal standards and raw outputs were normalized in MetaboAnalyst v5.0 (Figure S1).

#### 4.6.3. Statistical Analysis of Non-Targeted Metabolomics

Statistical evaluation of the non-targeted metabolomic data was conducted with the R-based statistical package MetaboAnalyst version 5.0 [34]. Important molecular features were elucidated in MetaboAnalyst using the multivariate analysis tools.

## 5. Conclusions

In conclusion, our results not only validate the classical understanding of sperm capacitation but also introduce novel insights into the metabolic shifts and regulatory effects of zinc during this crucial fertilization-capacity-endowing biological process. Using image-based flow cytometry and Gas Chromatography-Mass Spectrometry (GC-MS), we identified significant variations in sperm cells and in metabolite profiles across non-capacitated, capacitated, and zinc-supplemented capacitated spermatozoa.

Key findings include the depletion of primary energy substrates such as glucose, fructose, and citric acid during capacitation, indicating heightened energetic demands. This depletion was accompanied by increased levels of glucitol, lactic acid, and phosphate, suggesting that anaerobic glycolysis plays a role during capacitation events. The presence of extracellular zinc further modulated these metabolic pathways, as evidenced by elevated concentrations of glucitol, lactic acid, succinic acid, and fructose, alongside a reduction in saturated fatty acids like palmitic, dodecanoic, and myristic acids.

The observed metabolic changes underscore the dynamic interplay between mitochondrial oxidative phosphorylation and glycolysis during sperm capacitation, reflecting the sperm cell’s adaptation to varying energetic demands and confirming the previous literature. Zinc’s regulatory role is noteworthy, as it appears to influence both energy metabolism and fatty acid composition. The supplementation of zinc in capacitation media was solely used as a treatment to observe the regulatory effects of zinc on capacitation and should not be adopted for use in industry for capacitation-inducing media.

Future research should delve deeper into the functional consequences of these metabolic changes and the mechanistic underpinnings of zinc’s modulatory effects; it should also elucidate potential biomarkers for fertility outcomes in boars to offer more targeted breeding strategies and increase the maximal utilization of high-terminal-index, economically valuable sires by reducing the sperm per dose of high-fertility sires.

## Data Availability

The datasets presented in this article are not readily available because there is no public repository available for the data types generated in this study, including image-based flow cytometry files and lipidome data files. Requests to access the datasets should be directed to the corresponding author.

## Supplementary Materials

The supporting information can be downloaded at: https://www.mdpi.com/article/10.3390/ijms25147919/s1.
