# Supplementary material for: Metabolic Shift in Porcine Spermatozoa during Sperm Capacitation-Induced Zinc Flux
Source: Int J Mol Sci. 2024 Jul 19;25(14):7919. doi: 10.3390/ijms25147919 (PMC11276750; doi:10.3390/ijms25147919)
Supplement: Supplementary file 1 [file ijms-25-07919-s001.zip › Supplementary Data for Metabolism Manuscript.pdf]

Supplementary Data

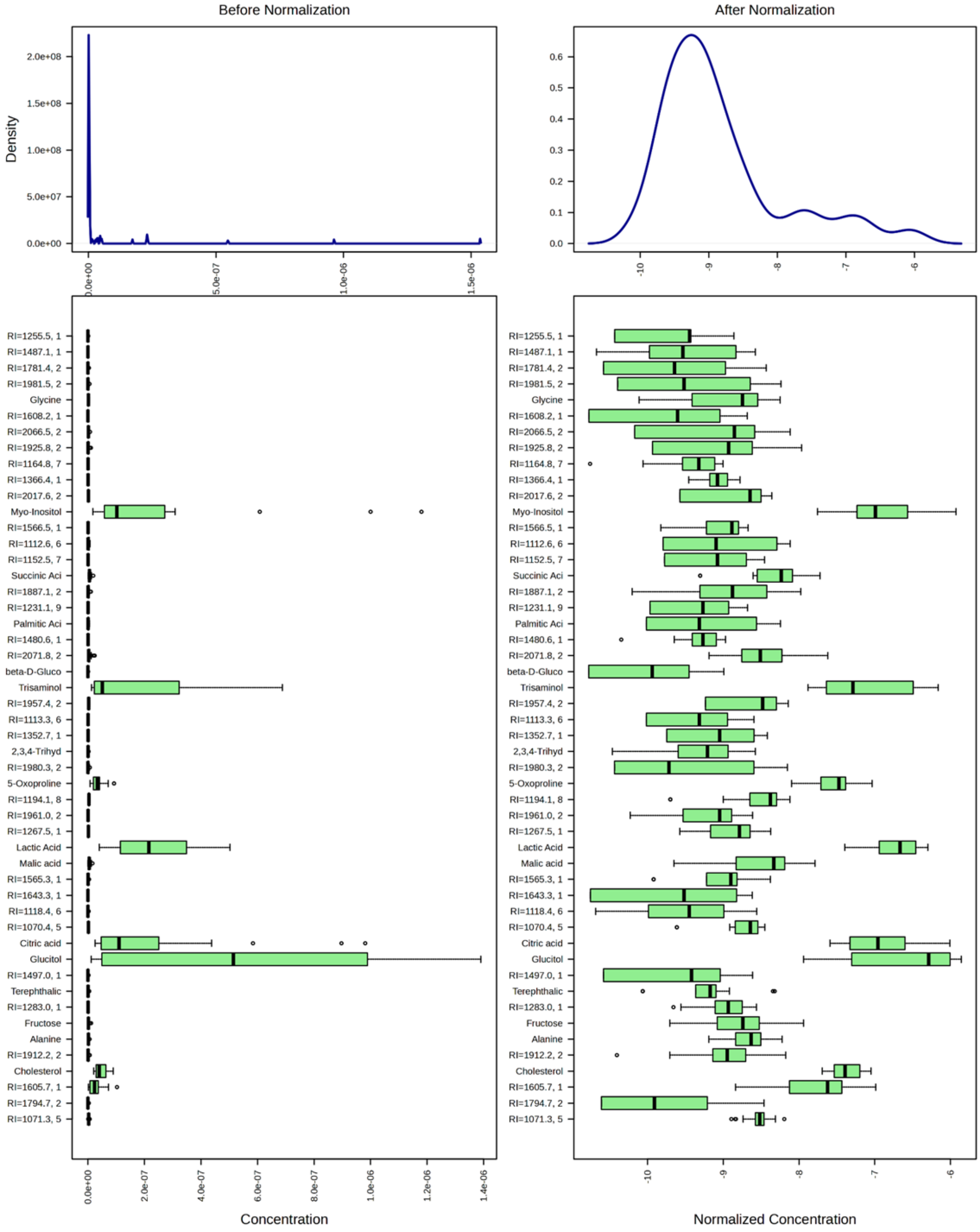

Figure S1. Normalization of the data was done through MetaboAnalyst 5.0 software [27]. Box plots and kernel density plots before and after normalization. The boxplots show at most 50 features due to space limit. The density plots are based on all samples. Selected methods: Row-wise normalization: N/A; Data transformation: Log10 Normalization; Data scaling: N/A.

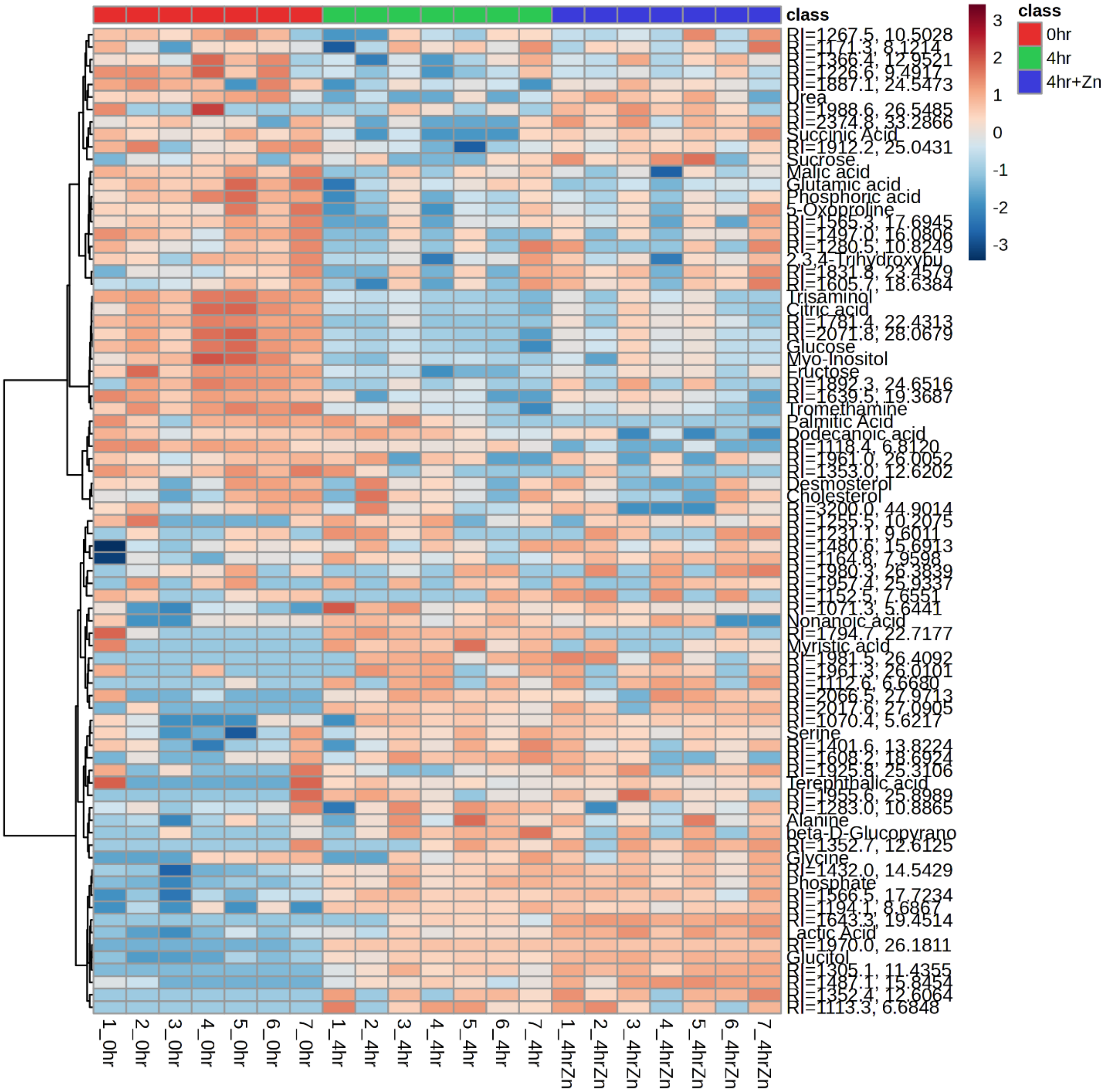

Figure S2. Heat Map Representing All 79 Metabolites in Sperm Metabolome of All Sires Individually Across All Treatments. Fold change illustrated by gradient from dark blue (negative) to dark red (positive). 0 hr (Non-Capacitated) are in red, 4 hr IVC are in green, and 4 hr IVC + 2mM zinc in blue for class. Metabolites starting with the name "RI=" are unidentified features that have been given a retention index-based name for tracking purposes.

Table S1. Percentages of Zinc signatures of each boar across all 3 treatments. Data obtained from the ImageStream^{X} MkII image-based flow cytometer.

Sample	Zn Signature 1%	Zn Signature 2%	Zn Signature 3%	Zn Signature 4%
1_0hr	0.4	91.6	7.6	0.4
2_0hr	1.8	97.5	0.6	0.1
3_0hr	0.3	96.0	3.3	0.4
4_0hr	1.3	96.5	1.5	0.7
5_0hr	0.9	87.6	9.9	1.6
6_0hr	4.5	95.1	0.4	0.0
7_0hr	1.5	97.3	0.8	0.4
1_4hr	27.2	27.6	45.2	0.0
2_4hr	10.7	8.9	79.1	1.3
3_4hr	22.4	9.1	66.5	2.0
4_4hr	5.9	26.6	67.5	0.0
5_4hr	24.4	9.4	66.2	0.0
6_4hr	36.5	10.2	52.4	1.0
7_4hr	35.6	19.5	44.3	0.6
1_4hr+Zn	11.5	29.7	58.6	0.3
2_4hr+Zn	10.7	25.0	63.3	1.1
3_4hr+Zn	18.8	45.1	34.7	1.4
4_4hr+Zn	14.4	51.0	33.5	1.2
5_4hr+Zn	35.3	33.4	29.9	1.4
6_4hr+Zn	55.6	32.3	11.7	0.5
7_4hr+Zn	68.6	14.3	16.0	1.1

Table S2. Percentages of PNA stained cells of each boar across all 3 treatments. Data obtained from the ImageStream^X MkII image-based flow cytometer.

Sample	PNA-%	PNA+%	PNA++%	PNA+++%
1_0hr	87.6	7.1	4.6	0.6
2_0hr	75.1	16.1	8.2	0.6
3_0hr	78.1	3.8	17.7	0.4
4_0hr	87.7	3.4	8.5	0.3
5_0hr	88.8	2.5	7.7	1.0
6_0hr	74.1	9.7	9.3	6.9
7_0hr	86.2	2.2	10.8	0.8
1_4hr	34.1	42.0	23.9	0.0
2_4hr	23.4	49.6	26.9	0.0
3_4hr	51.8	39.0	9.2	0.1
4_4hr	25.1	53.2	21.7	0.0
5_4hr	49.1	42.4	8.4	0.0
6_4hr	49.7	29.0	21.3	0.0
7_4hr	40.7	41.9	17.4	0.0
1_4hr+Zn	34.9	23.1	24.0	18.0
2_4hr+Zn	24.3	16.0	36.6	23.0
3_4hr+Zn	26.8	17.5	33.1	22.6
4_4hr+Zn	24.6	21.3	40.7	13.4
5_4hr+Zn	40.3	13.7	36.8	9.2
6_4hr+Zn	52.4	9.9	22.0	15.7
7_4hr+Zn	32.3	7.7	42.9	17.1

Table S3. Percentages of PI stained cells of each boar across all 3 treatments. Data obtained from the ImageStream^X MkII image-based flow cytometer.

Sample	PI-%	PI Transitioning%	PI+%
1_0hr	88.2	1.9	9.9
2_0hr	71.1	4.4	24.5
3_0hr	79.0	1.6	19.5
4_0hr	89.9	1.0	9.1
5_0hr	89.6	2.5	7.9
6_0hr	74.4	19.4	6.2
7_0hr	82.3	3.3	14.5
1_4hr	8.5	14.0	77.5
2_4hr	3.9	6.8	89.3
3_4hr	9.0	11.6	79.4
4_4hr	2.7	5.7	91.7
5_4hr	14.2	14.2	71.6
6_4hr	34.1	0.9	65.0
7_4hr	29.4	1.5	69.1
1_4hr+Zn	10.1	2.5	87.4
2_4hr+Zn	4.2	3.6	92.1
3_4hr+Zn	7.8	6.0	86.2
4_4hr+Zn	4.8	2.5	92.6
5_4hr+Zn	23.1	3.8	73.1
6_4hr+Zn	30.6	3.2	66.2
7_4hr+Zn	20.4	0.6	79.0
